# Self-organizing nanodot structures on InP surfaces evolving under low-energy ion irradiation: analysis of morphology and composition

**DOI:** 10.1186/1556-276X-9-403

**Published:** 2014-08-19

**Authors:** Tobias Radny, Hubert Gnaser

**Affiliations:** 1 Fachbereich Physik and Forschungszentrum OPTIMAS, Technische Universität Kaiserslautern, 67663 Kaiserslautern, Germany; 2 Institut für Oberflächen- und Schichtanalytik GmbH (IFOS), Trippstadter Str. 120, 67663 Kaiserslautern, Germany

**Keywords:** Nanodot structures, Ion irradiation, Atom probe tomography, InP, 79.20.Rf, 81.16.Rf, 68.35.Ct

## Abstract

Surfaces of InP were bombarded by 1.9 keV Ar^+^ ions under normal incidence. The total accumulated ion fluence Φ the samples were exposed to was varied from 1 × 10^17^ cm^−2^ to 3 × 10^18^ cm^−2^, and ion fluxes *f* of (0.4 − 2) × 10^14^ cm^−2^ s^−1^ were used. The surface morphology resulting from these ion irradiations was examined by atomic force microscopy (AFM). Generally, nanodot structures are formed on the surface; their dimensions (diameter, height and separation), however, were found to depend critically on the specific bombardment conditions. As a function of ion fluence, the mean radius *r*, height *h*, and spacing *l* of the dots can be fitted by power-law dependences: *r* ∝ Φ^0.40^, *h* ∝ Φ^0.48^, and *l* ∝ Φ^0.19^. In terms of ion flux, there appears to exist a distinct threshold: below *f* ~ (1.3 ± 0.2) × 10^14^ cm^−2^ s^−1^, no ordering of the dots exists and their size is comparatively small; above that value of *f*, the height and radius of the dots becomes substantially larger (*h* ~ 40 nm and *r* ~ 50 nm). This finding possibly indicates that surface diffusion processes could be important. In order to determine possible local compositional changes in these nanostructures induced by ion impact, selected samples were prepared for atom probe tomography (APT). The results indicate that APT can provide analytical information on the composition of *individual* InP nanodots. By means of 3D APT data, the surface region of such nanodots evolving under ion bombardment could be examined with atomic spatial resolution. At the InP surface, the values of the In/P concentration ratio are distinctly higher over a distance of approximately 1 nm and amount to 1.3 to 1.7.

## Background

Bombarding solid surfaces by energetic particles leads to a variety of phenomena that are closely correlated with the energy deposition processes of the incoming ions [1,2]. At the surface, ion irradiation may result in substantial morphological changes [3], resulting in a coarsening of the surface. Eventually, prolonged ion irradiation often leads to the development of a very specific surface topography. Interestingly, these structures can exhibit highly periodic features such as ‘nanodots’ [4,5] or ‘ripple’-like contours [6,7], with feature sizes in the nanometer range. These self-organized nanostructures evolving due to ion bombardment on surfaces have been studied quite thoroughly in the past decade [8-14]. Generally, the formation of these structures is assumed to be related to (and caused by) the interplay between ion erosion (which roughens the surface) and transport processes which induce a smoothing [9,10,12]; the latter could be effected by (beam-enhanced) surface diffusion [15,16] or viscous flow [17,18] within the ion penetration layer.

Theoretically, a substantial degree of understanding of ripple formation is due to the pioneering model proposed by Bradley and Harper (BH) [19] that considers the surface evolution in terms of such a dynamic balance between roughening and smoothing processes. The concept of BH combines the effects of sputtering and surface diffusion and is based on the sputtering theory of Sigmund [20,21]. The latter relates the rate of atom removal to the energy deposited by the incident ion in the near-surface region in a sequence of collisions. BH extended that approach and emphasized that the sputtering yield is proportional to the curvature of the surface; eventually, this may lead to a roughening. This process could be counteracted by surface relaxation processes. Combining these competitive mechanisms, BH derived an equation for the surface height *h*(*x*,*y*,*t*) [9,19]:

(1) $$\frac{\partial h}{\partial t}=-v_{0}+\frac{\partial v_{0}}{\partial \theta }\frac{\partial h}{\partial x}+\nu_{x}\frac{\partial^{2}h}{\partial x^{2}}+\nu_{y}\frac{\partial^{2}h}{\partial y^{2}}-K\nabla^{4}h$$

Here, *v*_0_ is the average erosion velocity of the surface which depends on the incidence angle of the ion beam *θ*, the ion flux, and the sputtering yield. ν_*x*_ and ν_*y*_ are functions of the ion beam parameters [9] and relate the sputtering yield at any point on the surface to the local curvature. The last term in Equation 1 represents surface diffusion of mobile species and is proportional to the second derivative of the curvature [22,23]. The parameter *K* depends on the surface energy, the diffusivity of mobile surface defects, and their average concentration. Such a diffusive process might also be triggered or enhanced by ion bombardment [8]. A similar functional form of smoothing can arise from ion-induced viscous flow in a thin surface layer [24,25]. Several extensions and modifications of the BH model were later envisaged [12,13,26].

Solutions of Equation 1 would predict that each Fourier component of the surface height will grow exponentially with a rate that depends on the wavevector, and a maximum growth rate might be reached. The corresponding modulation will outgrow the others and lead to ripples with a characteristic wavelength λ^*^:

(2) $$\lambda^{*}=2\pi \;\sqrt{2K/\nu_{\text{max}}}$$

where ν_max_ is the maximum of the two values ν_*x*_ and ν_*y*_ in Equation 1. The magnitude of the latter determines the orientation of the ripple pattern with respect to the ion beam direction [19].

For binary (or, more generally, multicomponent) specimens, the situation might be complicated by the potential presence of the preferential sputtering of one of the components [1,2]. This process will tend to modify the composition in a surface layer with a thickness of a few atomic layers for the low energies considered here. Relevant for the present context is the theoretical demonstration [27] that, apart from the formation of specific nanostructures (ripple or dots), compositional gradients may exist within individual of these features: for example, in ripple structures, one component will be enriched in the crests while being depleted in the valleys, and vice versa for the other component. Further theoretical approaches [28-31] confirmed and refined this possibility.

In a binary system A-B, *Y*_A_ and *Y*_B_ may denote the sputtering yields of species A and B (sputtered atoms per incoming ion). (*Y*_A_ and *Y*_B_ are not necessarily equal to the yields of the respective pure samples A or B.) If *Y*_A_ ≠ *Y*_B_, preferential sputtering will lead to steady-state *surface* concentrations *c*_s_ which deviate from the bulk concentrations *c*_b_ while the fluxes of emitted species should be proportional to their bulk composition for steady-state conditions [2]. As a consequence, a layer of altered composition *c*_s_ is formed near the surface. Its thickness Δ will amount to a few atomic layers for the low impact energies employed in this work. For planar specimens, such ion-induced surface modifications have been studied quite extensively in the past for a large variety of (binary) systems [1,2]. Typically, this surface layer is found to be enriched (depleted) in the species that has the lower (higher) sputtering yield. However, segregation (diffusion) processes may lead to rather abrupt concentration gradients at the surface, that is, *c*_s_ might not be constant over the depth Δ. In the presence of nanostructures, a height variation *h*(*x*,*y*,*t*) could be associated with a perturbation in composition, *ζ*(*x*,*y*,*t*) [27], where *ζ* = *c*_s_ − *c*_b_. Therefore, the ion-induced enrichment (depletion) might be site specific (e.g., different for crests or valleys in ripples) leading, eventually, to a modulation in composition that can be in or out of phase with the (ripple) topography. The evolution equations, to linear order in the perturbations, were shown to take the form [27]

(3) $$\frac{\partial \zeta }{\partial t}=A\nabla^{4}H+B\nabla^{2}\zeta -\mathrm{C\zeta}$$

(4) $$\frac{\partial H}{\partial t}=-A'\nabla^{4}H+B'\nabla^{2}\zeta +C'\zeta +D'\nu_{h}$$

where *H* = *h*/Δ and ν_*h*_ gives the slope and curvature dependence of the sputtering yield [19]. The coefficients of the terms on the right-hand sides of Equations 3 and 4 are specified in [27]. A key feature of this theoretical concept is the coupling between the height and composition modulations. An experimental examination of such correlated compositional modulations within individual nanostructures (ripples or dots) formed by ion bombardment would be required to verify that approach and to elucidate the pertinent processes. Because of the small dimensions, such an investigation is quite challenging and available data are rather limited.

In order to study such possible compositional variations in individual nanodots caused by ion bombardment, atom probe tomography (APT) has been used in this work. APT is a very unique analytical tool for the elemental characterization of solid materials on nanometer spatial scales [32,33]. In APT, ions are released via field evaporation from a tip with a very small radius of curvature (*R* less than approximately 50 nm) in the presence of a high electric field (approximately 30 to 50 V/nm). The removal of material from the tip releases atoms from continuously deeper layers of the specimen. A reconstruction of the complete data set provides ideally the original 3-dimensional distribution of the atoms in the analyzed sample volume. (A typical size would be 50 × 50 × 200 nm^3^). Several experiments have demonstrated that in APT analyses sub-nanometer spatial resolution can be achieved [32,33]. In fact, different types of nano-sized structures have been successfully analyzed by APT [34-36].

The objective of the present work was hence twofold: (i) to examine the formation and evolution of nanodots on InP surfaces under Ar^+^ ion bombardment and determine specific feature sizes (height, radius, and wavelength) as a function of irradiation parameters (ion fluence and ion flux) and (ii) to employ APT for a compositional analysis of *individual* nanodots with nanometer spatial resolution. This appears to constitute a completely novel approach of nanodot characterization.

## Methods

The experiments were carried out in a custom-built UHV apparatus which incorporates an electron-impact ion gun (IQ12/38, Leybold-Heraeus, Köln, Germany), a sample stage that can be translated in *x*-, *y*-, and *z*-directions and rotated in order to vary the ion-beam incidence angle, and a load-lock transfer system. Ion bombardment was done with Ar^+^ ions at normal incidence to the sample surface. The ion energy *E* was 1.9 keV and ion fluxes *f* of (0.4 − 2) × 10^14^ cm^−2^ s^−1^ were used. The total accumulated ion fluence Φ the samples were exposed to was varied from 1 × 10^17^ cm^−2^ to 3 × 10^18^ cm^−2^. All ion irradiations were carried out at room temperature.

The atomic force microscopy (AFM) measurements were done using a MFP3D (Asylum Research, Goleta, CA, USA) operated in contact mode, employing cantilevers (Veeco SNL-10, Plainview, NY, USA) with a nominal tip radius ≤12 nm. The AFM data were evaluated with the software package Gwyddion [37]. The radial autocorrelation function (ACF) was determined to derive the average separation (wavelength) *l* of the nanodots. Their dimensions (height *h* and radius *r*) were derived by employing a watershed algorithm [37].

Atom probe tomography (APT) was carried out in a LEAP 4000X HR instrument (CAMECA, Gennevilliers, France) which is equipped with a reflectron-type time-of-flight mass spectrometer and a pulsed UV laser (*λ* = 355 nm, pulse length approximately 10 ps). During the analyses (chamber pressure approximately 1 × 10^−11^ mbar), the specimens were cooled to temperatures in the range of 30 to 45 K. The laser pulse energy was 5 pJ at a repetition rate of 100 kHz. The mass resolution amounted to *M*/Δ*M*_FWHM_ ~ 1,000. The data reconstruction was done with the instrument's software package IVAS3.6.4. In APT, samples have to be in the shape of a tip with a very small radius of curvature. The preparation of such tips was done employing the cut-and-lift-out method [38], using an ALTURA 875 dual-beam focused ion beam (FIB) instrument (FEI, Hillsboro, OR, USA). To protect the thin surface layer of the specimen against destruction during cutting and milling, the samples were usually covered by an approximately 100-nm Cr-layer before FIB processing.

The specimens used in the present study were *n*-type InP(100) single-crystal wafers (Wafer Technology, Milton Keynes, UK). Before inserting them in the UHV chamber, they were cleaned ultrasonically in ethanol and distilled water and dried in a flow of nitrogen.

## Results and discussion

**Figure 1:**
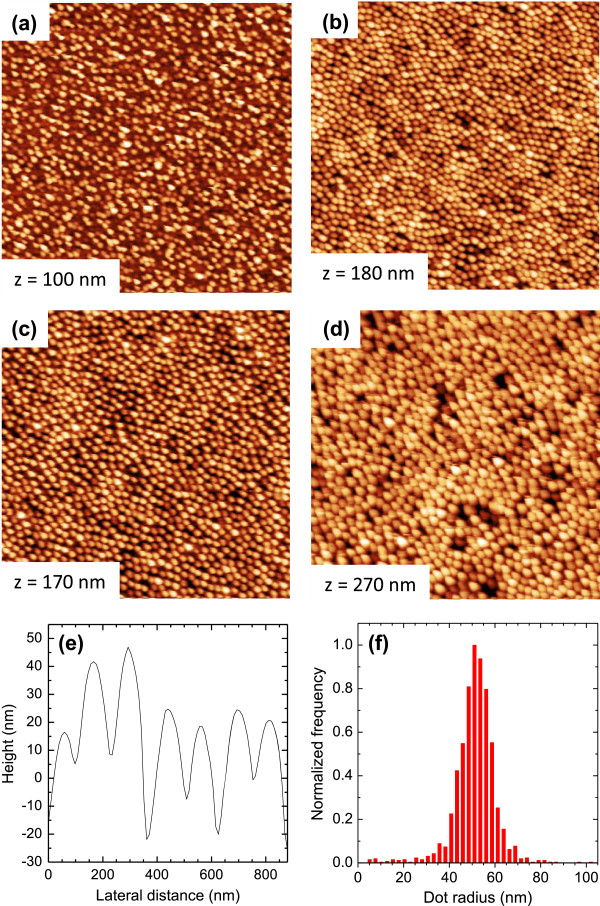
**AFM topographic images of InP surfaces bombarded by 1.9 keV Ar**^**+ **^**ions at normal incidence and an ion flux *f*** **= 1.5 × 10**^**14**^ **cm**^**−2**^ **s**^**−1 **^**with fluences of (a) 0.5, (b) 1, (c) 2, and (d) 3 × 10**^**18 **^**ions cm**^**−2**^**.** The size of the images is (5 × 5) μm^2^ and the height scale *z* is indicated. **(e)** Line profile across a selected part of the image shown in **(b)** displaying the cross sections of individual nanodots on the surface. **(f)** Normalized frequency distribution of the dot radius obtained for the image in **(b)**.

The main objective of the present work was the formation of regular nanodot structures on InP by ion irradiation and their compositional analysis by means of atom probe tomography (APT). Figure 1a,b,c,d shows four AFM images (5 μm × 5 μm) of InP surfaces bombarded by 1.9 keV Ar^+^ ions with fluences in the range of Φ = (0.1 − 3) × 10^18^ cm^−2^. The ion flux was *f* = 1.5 × 10^14^ cm^−2^ s^−1^. The images illustrate the presence of nanodots. Figure 1e displays a typical line profile across part of the image in Figure 1b, exemplifying the cross-sectional shape of individual nanodots. The normalized frequency distribution of the dot radius for that image is shown in Figure 1f, giving an average dot radius *r* = 52 ± 4 nm for this fluence (Φ = 1 × 10^18^ cm^−2^); a mean dot height *h* = 46 ± 13 nm and a lateral spacing *l* = 146 ± 18 nm were found from similar evaluations.

**Figure 2:**
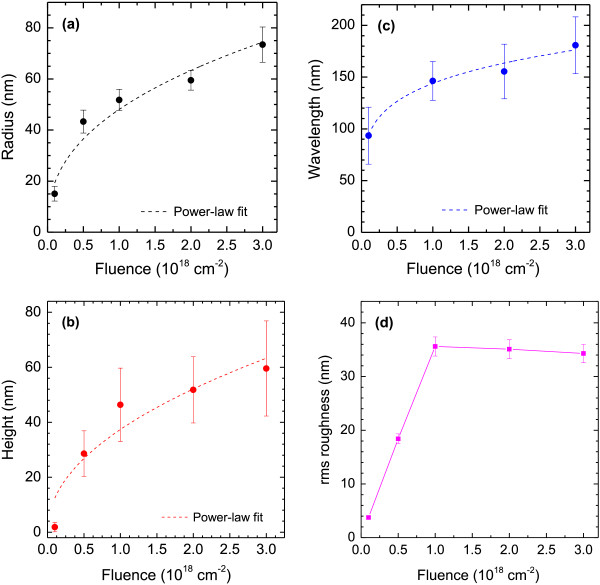
**The mean radius *r *(a), height *h *(b), and separation (wavelength) *l *(c) of the dots plotted as a function of the ion fluence Φ.** The error bars given in the plots represent the widths of the distributions of the respective values. The data are fitted by power-law dependences (dashed lines). **(d)** The rms-roughnesses of the corresponding AFM images. The ion flux was *f* = 1.5 × 10^14^ cm^−2^ s^−1^.

While for any given fluence, the dots exhibit fairly homogeneous distributions in terms of size (diameter and height) and mutual separation, these values are increasing distinctly with ion fluence Φ in the range investigated here (1 × 10^17^ cm^−2^ to 3 × 10^18^ cm^−2^). This finding is shown in Figure 2 for the dot radius *r* (a), the dot height *h* (b), the wavelength *l* (c), and the rms roughness (d) of the AFM images. (The error bars given in the plots represent the widths of the distributions of the respective values; see Figure 1f). It is noted that the radius derived for the smallest dot (*r* ~ 15 nm) could, however, be influenced by the size of the AFM tip (≤12 nm). The first three of these data sets can be fitted quite well by power-law dependences (dashed lines): *r* ∝ Φ^0.40±0.07^, *h* ∝ Φ^0.48±0.13^, and *l* ∝ Φ^0.19±0.02^. Such power-law functional relations have been observed in previous studies [12,13] but the respective exponents exhibit a wide variation and are usually determined by specific bombardment parameters such as the ion flux or others. The rms roughness, Figure 2d, is found to increase linearly with Φ up to Φ = 1 × 10^18^ cm^−2^ and remains roughly constant (approximately 35 nm) for higher fluences.

**Figure 3:**
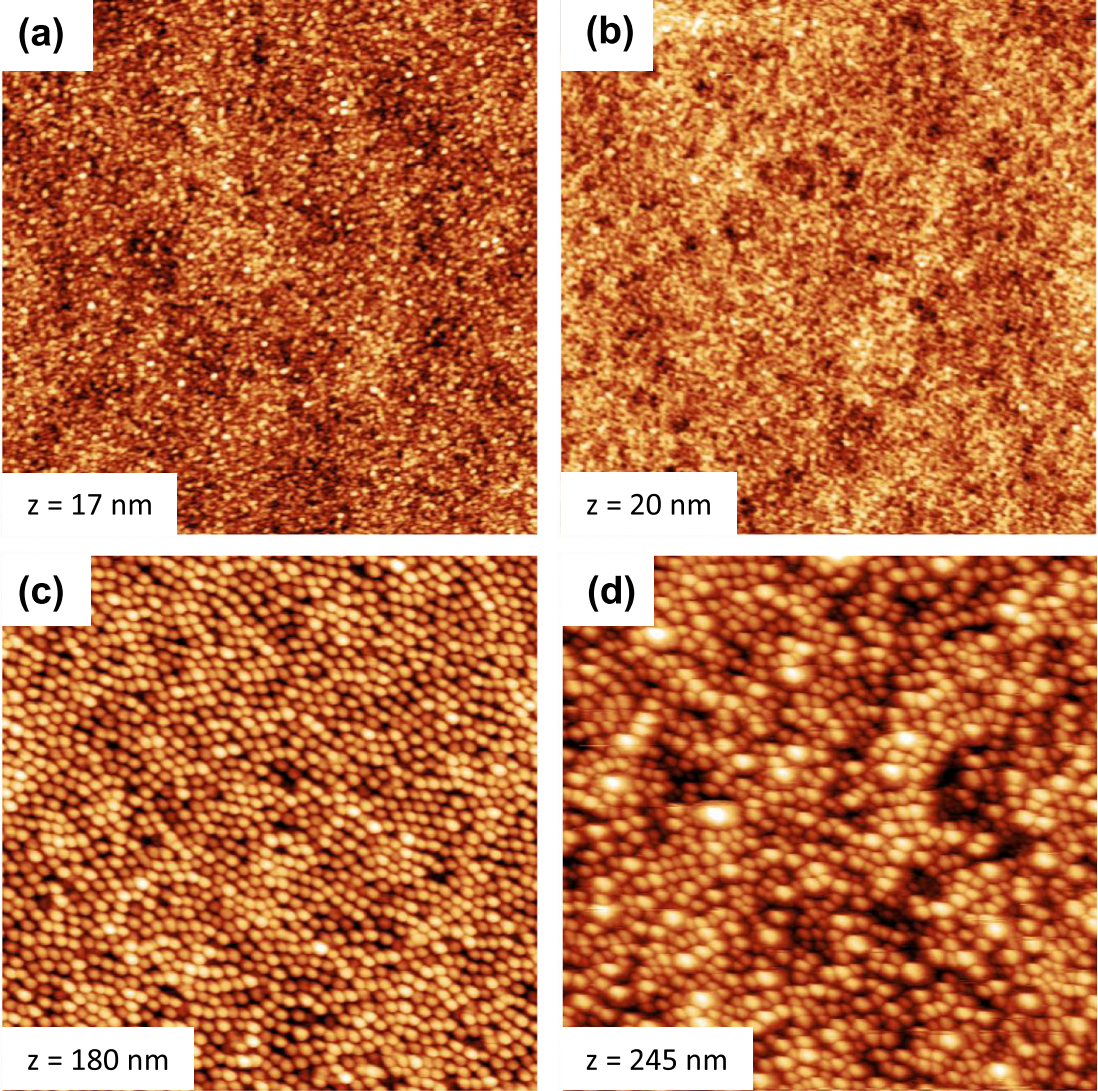
**AFM topographic images of InP surfaces bombarded by 1.9 keV Ar**^**+ **^**ions at normal incidence and an ion fluences of 1 × 10**^**18 **^**ions cm**^**−2 **^**with ion fluxes of (a) 0.4, (b) 1.1, (c) 1.5, and (d) 2.1 × 10**^**14**^ **cm**^**−2**^ **s**^**−1**^**.** The size of the images is (5 × 5) μm^2^ and the height scale *z* is indicated.

**Figure 4:**
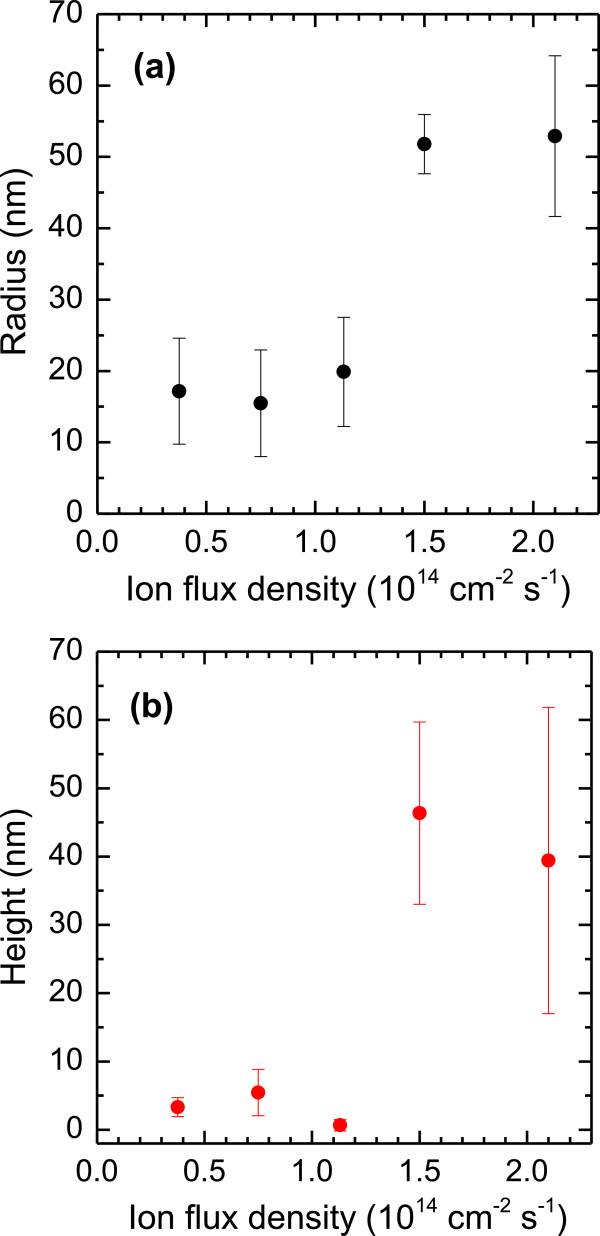
**The mean radius *r *(a) and height *h *(b) of the dots plotted as a function of the ion flux *f*.** The error bars given in the plots represent the widths of the distributions of the respective values. The ion fluence was Φ = 1 × 10^18^ ions cm^−2^.

Apart from the fluence-dependence, the nanodot dimensions exhibit also a rather distinct variation with the ion flux. Figure 3 shows AFM topographic images from InP surfaces bombarded with 1.9 keV Ar^+^ ions at four different values of *f*: 0.4 (a), 1.1 (b), 1.5 (c), and 2.1 × 10^14^ cm^−2^ s^−1^ (d). The ion fluence was Φ = 1 × 10^18^ cm^−2^ in all cases. The images illustrate qualitatively that the nanodot features are larger for the two higher ion fluxes as compared to the two lower ones. Figure 4 displays the mean dot radius (a) and dot height (b) derived from the AFM images as a function of the ion flux. The graphs confirm the rather abrupt increase of both values at a flux between 1.1 and 1.5 × 10^14^ cm^−2^ s^−1^. Radial ACF demonstrate furthermore that for *f* ≤ 1.1 × 10^14^ cm^−2^ s^−1^, no ordering of the dots exists. Such a threshold was observed also for 1-keV Ar^+^ bombardment of InP [39], but the corresponding value of *f* was somewhat lower (approximately 3.6 × 10^14^ cm^−2^ s^−1^). That study showed in addition that the formation of nanostructures on InP may depend on the sample temperature. This finding could imply that in Equation 1, the diffusive terms may be become dominant as compared to the erosive contributions.

Changes in the near-surface composition of InP caused by ion bombardment has been investigated over large surface areas (μm to mm) in several previous studies [2,40]. Typically, P was found to be depleted at the surface due to preferential sputtering; the In/P surface concentration ratio as determined from Auger electron spectroscopy amounts to approximately 1.7 for Ar^+^ ion energies of 1 to 5 keV [41] and no dependence on the Ar ion energy between 0.5 and 5 keV was found [42]. Apart from the preferential loss of P upon ion bombardment, the development of a pronounced surface morphology [43-45] and the formation of ripples and dots [5,39,46-48] was observed. In the present work, X-ray photoelectron spectroscopy (XPS) has been used to determine the surface composition of the bombarded sample, albeit over a larger area (approximately 1 mm). From these data, the In/P concentration ratio was found to be higher by a factor of 1.6 in the irradiated region as compared to the pristine InP surface, in agreement with the aforementioned studies.

**Figure 5:**
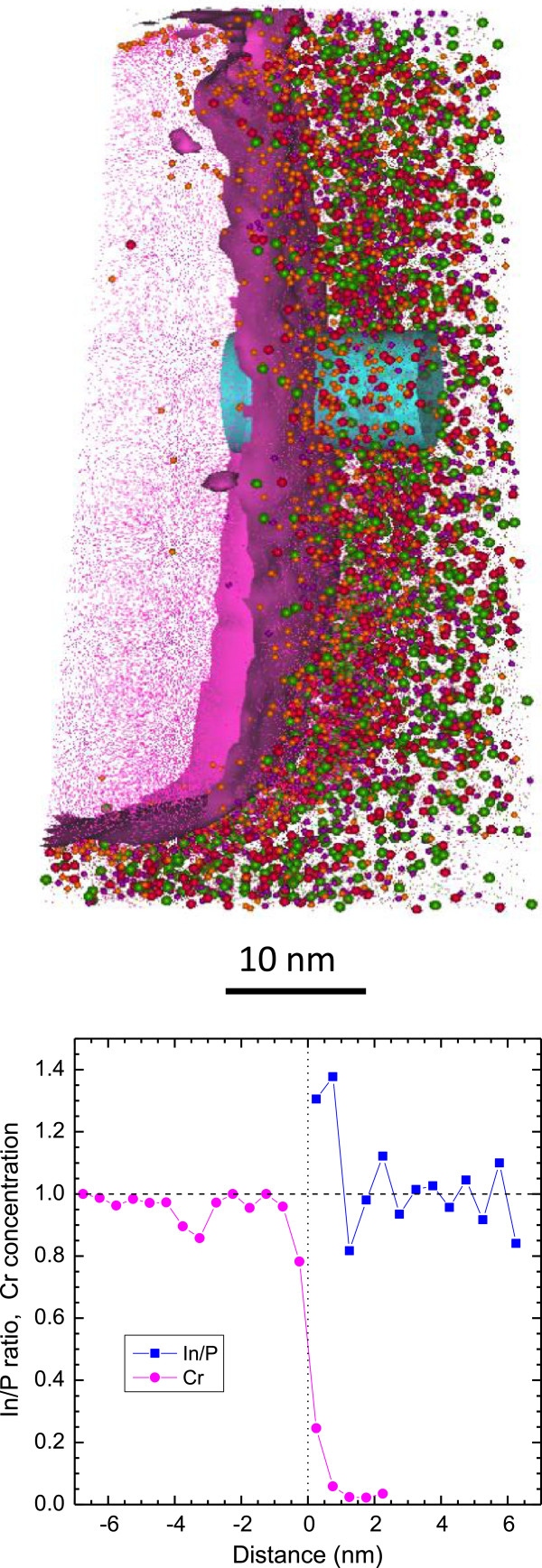
**3D APT sample volume of the interface region between the ion-bombardment InP surface of a nanodot and the deposited Cr layer (upper panel).** The analysis direction is from top to bottom. The colored dots represent different atoms in this sample volume: Cr (magenta), In (purple), P (orange), P_3_ (red), and P_4_ (green). In addition, an isoconcentration surface is depicted for Cr (the continuous magenta feature) which defines a volume with a Cr concentration >50% and separates the InP specimen proper and the deposited Cr layer. Also shown is a cylindrical region-of-interest (the cyan feature) which was used to derive a local concentration profile in this region (lower panel).

For the examination of possible compositional variations within individual nanodots by APT, tips were prepared from selected ion-irradiated InP samples. Because of their very small radius of curvature and the equally small dimensions of the nanodots, this fabrication process proved very delicate and only a limited number of tips were found suitable for APT analysis. Nonetheless, several 3D APT data sets revealed the interface region between the ion-bombardment InP surface of a nanodot and the subsequently deposited Cr layer. Figure 5 displays a 3D APT data volume showing such an interface region. The APT analysis direction is from top to bottom. Thus, the top region would correspond the original crest of the nanodot. The colored dots represent different individual ions detected from this sample volume: Cr (magenta), In (purple), P (orange), P_3_ (red), and P_4_ (green). In addition, an isoconcentration surface [33] is depicted for Cr (the continuous magenta feature) which encloses a volume with a Cr concentration >50% and separates the InP specimen proper and the deposited Cr layer. Also shown is a cylindrical region-of-interest (ROI, the cyan feature) located at that transition which was used to derive a local concentration profile. Two aspects of the APT analysis are of note: (i) An abundant emission of P_*n*_ cluster ions (with *n* ≤ 9) is observed. Although clustering has been found in APT of III-V semiconductors before [49,50], InP appears to constitute an extreme case in this respect. (ii) Despite the abundant cluster emission the In/P bulk composition of the specimens (i.e., away from any surfaces modified by ion bombardment) turns out to be correctly reproduced by APT, provided the laser pulse energy is 10 pJ or less. (A detailed discussion of these effects is, however, beyond the scope of the present paper and will published elsewhere).

A concentration profiles was determined using the cylindrical ROI (15 nm in length, 10-nm diameter) shown in the 3D data. This profile is given in the lower panel of Figure 5 and displays the In/P concentration ratio and the Cr atomic fraction as a function of distance along the cylinder with increments of 0.5 nm. Hence, each data point corresponds to a sample volume of 39.3 nm^3^. This and other profiles taken at different positions of the interface exhibit the abrupt decrease of the Cr concentration at the interface and a ratio In/P approximately 1 far beyond the interface. In fact, a mean ratio of In/P = 1.03 ± 0.10 is derived from a profile taken in the pristine InP (the region on the right-hand side of the 3D volume in Figure 5). On the other hand, close to the InP surface the values of In/P are distinctly higher over a distance of approximately 1 to 2 nm and amount to 1.3 to 1.7. This indicates a clear In enrichment at the surface but there appears to exist also some variation of this value depending on where the profiles are taken. The latter observation would support the theoretical proposal of composition changes correlated with topographical ones.

## Conclusions

The irradiation of InP surfaces by 1.9 keV Ar^+^ ions leads to the formation of nanodots. They exhibit little long-range order but their feature sizes such as height, diameter, and spacing show a distinct dependence on the fluence and the flux of the bombarding ions. The composition of individual nanodots was examined by atom probe tomography. However, a more thorough determination of possible compositional variations was found to be limited still by the very difficult preparation procedures of the tip specimens required for APT. It is envisaged nonetheless that ongoing and future experiments will solve these problems, enabling in this way an analysis of nanodot structures at an atomic resolution for various III-V semiconductor surfaces.

## Competing interests

The authors declare that they have no competing interests.

## Authors’ contributions

TR performed the ion-irradiation experiments and the AFM measurements and evaluated these data. HG carried out the atom probe analyses and the related data evaluation, and wrote the manuscript. Both authors read and approved the final manuscript.
